# The Connection Between the Nervous System and Machines: Commentary

**DOI:** 10.2196/16344

**Published:** 2019-11-06

**Authors:** Giacomo Valle

**Affiliations:** 1 The Biorobotics Institute Sant'Anna School of Advanced Studies Pisa Italy; 2 Translational Neural Engineering Lab École Polytechnique Fédérale de Lausanne Lausanne Switzerland

**Keywords:** brain-machine interfaces, neural electrodes, neural recording, neurostimulation, sensory-motor dysfunctions

## Abstract

Decades of technological developments have populated the field of brain-machine interfaces and neuroprosthetics with several replacement strategies, neural modulation treatments, and rehabilitation techniques to improve the quality of life for patients affected by sensory and motor disabilities. This field is now quickly expanding thanks to advances in neural interfaces, machine learning techniques, and robotics. Despite many clinical successes, and multiple innovations in animal models, brain-machine interfaces remain mainly confined to sophisticated laboratory environments indicating a necessary step forward in the used technology. Interestingly, Elon Musk and Neuralink have recently presented a new brain-machine interface platform with thousands of channels, fast implantation, and advanced signal processing. Here, how their work takes part in the context of the restoration of sensory-motor functions through neuroprostheses is commented.

Significant research in biology, medicine and engineering has sought to obtain effective solutions to improve quality of life of human subjects affected by sensory-motor disorders. Neuroprosthetics are implantable devices designed to replace or improve the function of a disabled part of the nervous system [1]. This technology is relatively recent, as the first neuroprosthetic device successfully implanted was a cochlear implant in 1957 [2]. Since then, such an approach has been expanded to many different applications, which include motor prosthetics [3-6], sensorimotor prosthetics [7-9], visual prosthetics [10,11], and cognitive prosthetics [12].

Up till now, patients who used brain-machine interfaces have had a quite poor perception of the instantaneous behavior, position, or motion of the robotic device, which has prevented them from operating in fully closed-loop and natural control. The restoration of sensory feedback and voluntary control, along with the development and successful integration of these sensor modalities, is a mandatory step towards the realization of future bidirectional neuroprostheses [13].

The challenges described above can be addressed by creating a brain-machine interface that utilizes the processing power of the human brain to control the robotic device. Directly connecting to the human nervous system means closing the gap between user intent and the expected behavior of the apparatus. Furthermore, generating a shorter loop between user intent and device behavior or motion (by eliminating part of the low-level sensor-based control) will allow for easier control, a reduced learning investment, and a reduced cognitive burden of operating the device.

Neural interfaces play a pivotal role in the efficacy of a neuroprosthetic. Due to their ability to read out electrical activity from the nervous system, it is possible to decode signals into cognitive, sensory, or motor information through the use of computational methods. This information can then be used to control a prosthetic device, robot, or computer. It also induces better understanding of brain behavior through the recording of neural activity, providing information about sensory areas responsible for hearing or sight (sensory prosthetics), or helping to regulate malfunctioning motor functions (motor prosthetics). On the other hand, pacemaker or bladder control neuroprosthetics also use similar physical principles, targeting the autonomic nervous system and helping patients with paraplegia due to spinal cord damage [14].

In a recent article, Elon Musk and his company Neuralink presented a new platform to target the brain for neuroprosthetic applications [15]. They used arrays of small and flexible electrodes (called threads), with 3072 electrodes per array, distributed across 96 threads. They also developed a neurosurgical robot able to insert six threads (192 electrodes) per minute. Each thread can be individually inserted into the brain with high precision, avoiding surface vasculature and targeting specific brain zones. The electrode array is packaged into a small implantable device that contains custom chips for low power, onboard amplification, and digitization. Moreover, since neural spikes in a brain-machine interface must be detected in real time to maximize decoding efficacy, Neuralink has developed a custom online spike-detection software that has achieved a spiking yield of up to 70% in chronically implanted electrodes. Musk’s long-term idea consists of enabling humans to connect their brains to machines, and Neuralink’s approach to a brain-machine interface has shown unprecedented packaging density, extensibility, and scalability in a clinically relevant package. The main properties of the neural electrodes are related to their biocompatibility, long-term stability, and recording/stimulating selectivity when interfacing with both peripheral and central nervous systems [16,17]. Therefore, more tests should be performed for a complete validation of this new platform. This step is not trivial, as it is crucial to show the possible translation of this approach to humans. Further, it is necessary to demonstrate the effective benefits of using this new technology in comparison to other techniques that have been widely tested in the previous decades. The hypothetical complete brain-machine connection has become a closer possibility, but it is not ready just yet.

In this field, many devices and smart materials have been presented as effective solutions to interfacing with nervous tissues, enabling an intimate connection between the brain and machines in animals and even in humans [18]. Understanding how to interact with the brain using advanced algorithms has become of great clinical interest now, both to decode neural information [19] and to encode natural sensations by exploiting biomimetic neurostimulation strategies [20]. Moreover, advanced data processing methods have to be developed to bring these technologies to real life application. In this direction, new tools like machine learning and quantum computing will help to bring this concept to reality.

In the near future, neurotechnologies will continue to grow. More accurate and advanced computer simulations (eg, computational modelling) will allow researchers to test and validate these technologies even quicker. Implantable neurotechnologies will literally become part of us. Direct bidirectional communication between the brain and external devices, the transformation that this connection brings about, and the blurring of the boundaries between humans and machines, are issues that raise several ethical, social, and cultural concerns. Personal identity, physical integrity, and the human dignity [21] of people using the next generation of brain-machine interfaces will surely require further attention.
